# The Impact of Acute Aerobic Exercise on General and Food-Related Inhibitory Function Among Young Adults with Obesity: An Event-Related Potential (ERP) Study

**DOI:** 10.3390/brainsci15010059

**Published:** 2025-01-10

**Authors:** Chun Xie, Tao Huang, Yingying Wang, Peisi Wang, Yanxia Chen, Jiali Qian, Guozhuang Chen, Kun Wang

**Affiliations:** 1 Department of Physical Education, Shanghai Jiao Tong University, Shanghai 200240, China; xiechun_628@163.com (C.X.); taohuang@sjtu.edu.cn (T.H.); wps0818@sjtu.edu.cn (P.W.); yanxia1201@sjtu.edu.cn (Y.C.); qianjiali@sjtu.edu.cn (J.Q.); chenguozhuang@sjtu.edu.cn (G.C.); 2 School of Psychology, Shanghai University of Sport, Shanghai 200438, China; wangyingying@sus.edu.cn

**Keywords:** obesity, acute aerobic exercise, inhibitory function, food, N2, P3

## Abstract

**Backgrounds and Objectives**: Obesity presents a significant global public health challenge and is associated with declines in both general and food-related inhibitory control, crucial for maintaining a healthy weight and preventing obesity progression. An increasing body of research suggests that acute aerobic exercise may improve inhibitory function. However, the effects and underlying mechanisms of acute aerobic exercise on both general and food-related inhibition in obese adults remain unclear. This study aimed to explore the potential impacts and underlying neuroelectronic mechanisms of a single session of aerobic exercise at varying intensities on general and food-related inhibitory functions among young adult males with obesity. **Design**: A within-subject design comprising three sessions (control, low-intensity exercise, moderate-intensity exercise) × three picture types (high-calorie food, low-calorie food, neutral picture) was employed. **Methods**: Eighteen young adult males with obesity [body mass index (BMI): 34.60 ± 4.21 kg/m^2^, aged 24.50 ± 5.13 years (Mean ± SD)] were recruited. They participated in three intervention sessions: acute aerobic exercise at low [40–50% maximal Heart Rate (HR_max_)], moderate (65–70% HR_max_), and a control session (sitting rest), separated by five-day intervals in a counterbalanced order. Following each session, participants performed a food-related Go/No-go task, and EEG recordings (N2 and P3 components) were conducted within 15 min. **Results**: Moderate-intensity exercise elicited larger N2 amplitudes compared to the control session across different picture types and task conditions. However, there was no significant effect on behavioral indicators or P3 amplitude across sessions. Additionally, food stimuli (both high- and low-calorie) resulted in lower No-go accuracy and smaller N2 amplitudes compared to neutral stimuli. **Conclusions**: Acute moderate-intensity exercise might influence general and food-related inhibitory function in obese individuals at the neuroelectric stage, potentially by enhancing attentional resources for managing cognitive control and conflict detection. Moreover, reduced N2 amplitudes and No-go accuracy in response to food stimuli compared to non-food stimuli indicate a diminished ability to allocate attentional and neural resources to manage food-related conflicts. However, due to the relatively small sample size, caution is advised when generalizing these findings to the broader population. The pilot test indicated that obese participants had difficulty sustaining high-intensity exercise at 80–90% of their maximum heart rate for a continuous or 20 min period, highlighting potential challenges in exercise adherence at high intensities within this population. Future research is needed to utilize interdisciplinary approaches and multimodal technologies to clarify how exercise influences food-related cognition, appetite regulation, and brain mechanisms in obesity, aiming to better contribute to the prevention and treatment of obesity.

## 1. Introduction

Obesity poses a significant public health issue globally [1,2] and is linked to cognitive decline. Extensive studies including cross-sectional and meta-analyses, have shown that obesity not only exhibited deficits in general cognitive function [3,4,5,6] but also in food-related cognitive function (especially in food-related inhibitory function) [7,8,9,10], which is critical for sustaining a healthy weight and impeding the acceleration of weight gain [8,11]. Furthermore, previous studies have shown that obese individuals exhibit atrophy in the prefrontal cortex (PFC), which is responsible for food-related cognitive control [10], diminished activation of the PFC [12,13], and decreased P3 or N2 amplitudes in food-related inhibitory tasks, highlighting the difficulty in utilizing accessible resources for effective cognitive control towards food [14,15,16]. Therefore, there is a need to pursue targeted treatments aimed at enhancing both general and food-specific inhibitory control in individuals with obesity.

Numerous studies have found that acute aerobic exercise could facilitate cognitive function and inhibitory function in healthy-weight individuals [17,18,19,20,21]. However, it remains unclear whether these benefits extend to individuals with obesity in general and in food-related cognitive function, as current findings are still limited and present a mixed picture. For example, Raine et al. [22] reported severe obesity might not entirely benefit from a single bout of aerobic exercise as they observed higher BMI individuals exhibiting diminished cognitive effects. Vincent and Hall [23] and Wen and Tsai [24] found no change in inhibitory function among overweight and obese adults following acute aerobic exercise or aerobic combined resistance exercise. However, Flack et al. [25] demonstrated that acute aerobic exercise could enhance the cognitive function related to food in overweight and obese adults. Zhang et al. [26] reported improved general and food-specific inhibitory control among obese adolescents following a moderate-intensity acute exercise intervention. Hence, it is necessary to investigate further whether acute aerobic exercise can confer cognitive benefits for adults with obesity and elucidate the specific brain mechanisms involved.

Event-related potentials (ERPs) offer high temporal resolution and are frequently employed to elucidate the precise brain mechanisms of general cognition [17,19,20,27,28] and food-related cognition among individuals with overweight and obesity [29,30,31] influenced by acute exercise. The P3 component is commonly utilized to explore inhibitory control processing and attentional resource allocation [32,33], whereas the N2 component is typically regarded as indicative of sensory processing and the monitor of conflict [32,34]. Some studies observed decreased N2 and increased P3 amplitude after acute moderate-intensity aerobic exercise (MIE) [19] but others reported increased N2 and unchanged P3 amplitude in normal-weight individuals [35]. While ERP findings regarding general and food-related inhibitory function across different intensities of aerobic exercise in obese individuals are scarce in the literature, a few studies have presented valuable evidence. Wen and Tsai [24] detected a decreased N2 and increased P3 amplitude in a general inhibitory task following a single bout of aerobic combined-resistance exercise among obese women. Bailey et al. [11] focused on healthy-weight adults; they found an acute aerobic exercise at 70% VO_2max_ could elicit larger N2 and P3 amplitudes in food-related inhibitory control processing than the 35% VO_2max_ and the control session, which indicated an enhanced and more efficient engagement of inhibitory and cognitive control related to food. Additionally, the previous study found that a single bout of high-intensity interval exercise could increase the P3 and late positive potential (LPP) amplitudes in a food-related Flanker task in adults with obesity [36]. Therefore, given the different results and relatively limited evidence about N2 and P3 components in this field, further investigation into these two components induced by general and food-specific cognitive function following acute aerobic exercise in obesity is warranted.

The study aimed to explore the potential impacts of a single bout of aerobic exercise on both general and food-related inhibitory function among obese young male adults and the associated neuroelectronic mechanisms. Previous research has often utilized within-subject designs to investigate how acute exercise affects cognition and event-related potential (ERP) metrics [25,35,36,37,38,39,40]. In these designs, each participant acts as their own control, thereby reducing the impact of individual differences. This approach facilitates effective comparisons with a relatively small sample size. Additionally, considering potential issues such as excessive sweating during the exercise intervention following EEG cap placement for pre-exercise testing, which could cause EEG paste to flow and disrupt electrode connectivity, our experimental design focuses on comparing post-intervention effects, rather than pre-and post-test comparisons to compromise the accuracy of EEG recordings. A within-subject design comprising three sessions (control, low-intensity exercise, moderate-intensity exercise) × three picture types (high-calorie food, low-calorie food, neutral picture) was employed. Additionally, considering prior studies that have identified differences in food cravings, cognitive abilities, and neural reactions to food stimuli across genders [41,42], this study focused solely on male participants to avoid potential gender-based variations [43]. It was hypothesized that a single session of aerobic exercise could improve both general and food-related inhibitory control in individuals with obesity and result in changes in N2 and P3 amplitudes. It is proposed that this neuroelectronic process involves the engagement of attentional resources to enhance inhibitory control.

## 2. Methods

### 2.1. Participants

Participants were recruited from an obesity camp, with assistance from camp managers and coaches for recruitment purposes. Participation was voluntary, with 25 young male adults between the ages of 18 and 35 initially enrolled from Shanghai, China. G*Power 3.1 software was utilized to conduct the power calculation [44]. The sample size for the study was determined based on Ligeza et al. [35], who employed a within-subject design to investigate the effect of acute exercise on inhibitory function. In their study, Ligeza et al. [35] reported an effect size (*η*^2^*_p_*) of 0.22 and 0.36 for the main session effect on reaction time and N2 amplitude, respectively, with a sample size of 18 subjects. The calculations indicate that to achieve a power of 0.80 and a significance level of 0.05 in a within-subject design with three repeated measures per session, at least eight participants are necessary to detect a main session effect size of 0.22 or larger. Furthermore, at least five participants are required to detect a main session effect size of 0.36 or greater. All participants fulfilled the following requirements: (a) a body mass index (BMI) of 28 kg/m^2^ or more (as per China’s obesity threshold) [45,46,47]; (b) an absence of endocrine or cardiovascular issues; (c) an absence of mental health concerns; (d) no significant illnesses; (e) a lack of recent drug or substance use for weight loss impacting blood sugar and fat metabolism; (f) no exercise restrictions in line with the Physical Activity Readiness Questionnaire [48]; (g) standard or corrected vision sharpness; and (h) dominance of the right hand. Every participant gave their informed agreement, which was sanctioned by the Shanghai University of Sport Ethics Committee (#102772019RT005). Following preliminary screening, two participants with hypertension on antihypertensive medication were excluded, resulting in a sample of 23 qualified participants. During the experiment, five participants withdrew from the camp due to personal reasons and did not complete all sessions, leaving 18 participants who completed all tests and were included in the data analysis. The demographic characteristics of these participants are presented in Table 1.

### 2.2. Food-Related Go/No-Go Task

**Figure 1:**
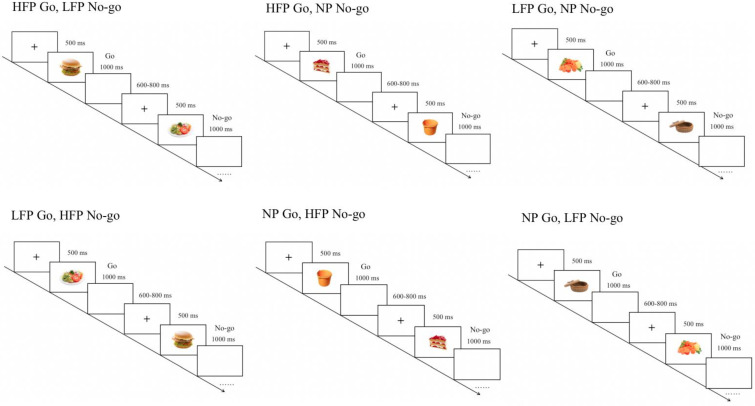
Food-related Go/No–go task paradigm (six blocks presented in a randomized order). HFP = high-calorie food picture; LFP = low-calorie food picture; NP = neutral picture.

E-Prime 2.0 software was utilized to design the food-related go/no-go task to assess the general and food-related inhibitory control. Initially, a black cross-fixation appeared on a white background for 500 ms, succeeded by the display of a stimulus image at the center of the screen, covering half of its dimensions. Participants were instructed to promptly respond by pressing the number “5” key when presented with the go stimuli. After the pressing, the stimulus vanished, and a white blank screen appeared for a random duration of 600–800 ms. The stimulus had a maximum presentation duration of 1000 ms, with no response deemed as an error. Participants were instructed to withhold key responses when the stimulus type was a no-go. A correct response to the stimulus was recorded if it persisted for 1000 ms before transitioning to the blank screen; otherwise, it was considered erroneous. The experiment comprised six blocks, which were presented in a randomized order. Each of the two types of high-calorie food images, low-calorie food images and neutral images, were used as stimuli for both go and no-go conditions, resulting in six distinct task blocks. Participants completed a practice block before each formal experimental block, consisting of 12 trials with a go-to-no-go ratio of 2:1. Stimuli were randomized during the presentation. Participants received feedback on reaction time and accuracy after each trial, presented on the blank screen. Once the accuracy reached 80% or higher, participants proceeded to the formal experiment. Each formal experimental block included 120 trials, maintaining the same go-to-no-go ratio as the practice block. The procedure of the experimental paradigm is illustrated in Figure 1. All of the food stimuli were selected from a standardized food picture database [54] and the same as that employed in the published paper [16]. The standardization process for these images involved several procedures [54]. Each image is a color photograph with a resolution of 600 × 450 pixels (96 dpi, sRGB color format) and a consistent white background. To ensure uniformity, the images were selected and edited with attention to factors such as viewing distance (approximately 80 cm), angle, and figure–ground composition. Matlab R2011b (The MathWorks, Inc., Natick, MA, USA) and a Canny edge detection algorithm (Canny, 1986) were then used to standardize various image characteristics, including color, size, brightness, within-object contrast, spatial frequencies, and complexity. The study encompasses 180 stimulus images, distributed among three categories: high-calorie foods, low-calorie foods, and neutral images, each comprising 60 images. High-calorie food images included pictures of fried foods and desserts, such as a hamburger, French fries, chocolate cookies, and strawberry cake. Low-calorie food images featured vegetables and vegetable-based salads, including images of a salad plate, tomatoes, veggie mixes, and cucumbers. Neutral images were selected to represent neutral images commonly found in daily and office settings, such as a bucket, clock, books, and sofa. Each image was presented four times. Image numbers used in the task are presented in Appendix A.

### 2.3. Electrophysiological Recording and Analysis

EEG signals were recorded by a 64-channel recording and analysis system (Brain Products GmbH, Munich, Germany). Ag/AgCl electrodes were affixed to a 64-channel cap following the international 10–20 standard system for EEG electrode placement and signal acquisition. FCz was employed as the reference electrode, while AFz served as the ground electrode during data collection. Additionally, an electrode was placed 1 cm lateral to the right eye for horizontal electrooculography (HEOG) recording, and 1 cm below the right eye in the infraorbital area for vertical electrooculography (VEOG) recording. Signals were collected from 34 electrode sites to optimize efficiency, including Fz, Cz, Pz, Oz, CPz, POz, F1, F2, F3, F4, C1, C2, C3, C4, P1, P2, P3, P4, O1, O2, FC1, FC2, FC3, FC4, CP1, CP2, CP3, CP4, PO3, PO4, TP9, TP10, as well as HEOG and VEOG. This setup facilitated the application of the gel and ensured stable electrode connections within approximately 15 min after exercise, enabling high-quality EEG data acquisition. Electrode impedance was maintained below 10 kΩ and with continuous digitization at 1000 Hz throughout the session.

ERP data were recorded using Brain Vision Recorder software 2.0 and analyzed with Brain Vision Analyzer 2.0. During data processing, we re-referenced to the average of potentials at TP9 and TP10 initially, and the remaining channels were used as an input for ICA, ensuring the full rank of the data [55]. Out of a total of 48 datasets (18 participants, each contributing three sessions), seven instances of bad P2 channels were identified. These channels were corrected using topographic interpolation, specifically employing the spherical spline interpolation method. To eliminate interference noise at 50 Hz from the electricity network, a notch filter from the IIR filters set in Brain Vision Analyzer 2.0 was applied, specifically tuned to the 50 Hz frequency. Subsequently, we applied low-cutoff and high-cutoff filters, respectively. The low-cutoff filter was set with a cutoff frequency of 0.1 Hz and a slope of 24 dB/octave, while the high-cutoff filter was configured with a cutoff frequency of 30 Hz and the same slope of 24 dB/octave. The independent component analysis (ICA) embedded in Analyzer 2.0 was utilized to remove eye blinks. Detailed procedure and parameters can be accessed in the manual, available here: https://www.brainproducts.com/downloads/manuals/, accessed on 6 January 2025. Then, data were included if the response was correct and were segmented into epochs, ranging from 200 ms before to 1500 ms after stimulus onset, totaling 1700 ms. Automated artifact rejection excluded all epochs with the amplitudes exceeding ±100 μV. Subsequently, a 200 ms prestimulus window was used for baseline correction. Finally, ERPs elicited under different conditions were averaged separately. The mean numbers of segments by sessions, task conditions, and picture types are presented in Table 2. Analyses focused on N2 and P3 area-average amplitudes, with time windows of N2 (200–300 ms), and P3 (350–600 ms) after stimulus onset at the Cz (N2) and Pz (P3) electrode site, respectively [56].

### 2.4. Experimental Procedure

The study involved multiple sessions conducted over approximately two months. Each eligible participant visited the laboratory three times, with each session scheduled at the same time, one hour after a meal, to minimize the influence of dietary intake on exercise performance and test results. Participants engaged in three experimental conditions across these visits: a control session (no exercise), a moderate-intensity exercise session, and a low-intensity exercise session. The order of sessions was counterbalanced to mitigate potential order effects, and a five-day interval was maintained between sessions to ensure a sufficient washout period and minimize practice effects. During their first visit, participants reviewed and signed the informed consent form, completed a demographic survey, and underwent the following assessments. Height (TANITA, Tokyo, Japan) and weight (Yaohua Weighing System Co., Shanghai, China) were measured to evaluate BMI [BMI (kg/m^2^) = weight (kg)/height (m)^2^], while eating styles were assessed by the Dutch Eating Behavior Questionnaire (DEBQ) [53] to characterize the study participants [50,51]. Extensive validation efforts have established the questionnaire’s reliability (Cronbach’s alphas > 0.79), applicable to both individuals of normal weight and those with obesity [57]. Additionally, cardiorespiratory fitness (VO_2peak_) was assessed using the YMCA submaximal ergometer exercise assessment [37,49] following the control session. This assessment is recognized for its safety and suitability for individuals with obesity. The stationary cycle (MONARK 894E, Vansbro, Sweden) was employed in the exercise experiment to reduce the likelihood of knee hurts from running in obesity [58,59]. Two exercise intensities (low and moderate) were selected specifically for obese participants to ensure the experiment’s feasibility and prioritize their safety. Given the heavier weight burden of individuals with obesity in the present study, these exercise intensities were chosen to avoid higher exercise volumes, which could result in higher exercise volumes if similar intensities were applied to healthy-weight individuals.

Each laboratory visit followed a standardized procedure, designed to ensure consistency and minimize variability in external factors. Each session lasted approximately 1.5 h, from arrival to departure, and consisted of three phases: preparation, experimentation, and post-experiment testing. Preparation phase (15 min): Upon arrival, participants were greeted and provided with a brief overview of the session’s objectives and procedures. They were guided to a preparation area where baseline physiological measurements, including heart rate and blood pressure, were assessed. A Polar heart rate monitor (FT1, Polar Electro Oly, Finland) was secured below the chest, and its placement was verified by the experimenter to ensure accurate readings. Experimental phase (30 min): depending on the assigned condition for the session; low-intensity exercise or moderate-intensity exercise; participants performed the formal exercise intervention, which included a 5 min warm-up, followed by a 20 min aerobic exercise session [low-intensity exercise session aiming for a heart rate between 40–50% of the person’s maximal heart rate (HR_max_) and moderate-intensity exercise session aiming for 65–70% HR_max_] (Norton et al., 2010) [60], and ended with a 5 min cool-down phase. During the intervention, a heart rate monitor was used to measure the HR every two minutes. Control session: Participants were seated comfortably in a quiet, temperature-controlled room. Participants experienced a matched period of sedentary rest, where they were instructed to remain seated quietly to avoid introducing stress or significant mental stimulation. Post-Exercise Assessment (45 min): Immediately after the experimental phase, the subjects were required to dry their hair using a hairdryer as soon as possible to reduce sweat’s effect on EEG recording. Then, the food-related Go/No-go task and EEG recordings were carried out within 15 min following each experimental phase.

### 2.5. Statistical Analysis

Using E-Prime 2.0, Go reaction time (Go RT), Go ACC, and No-go ACC across different sessions and picture types of food-related Go/No-go task were collected. For the behavior data, MATLAB software was adopted to preprocess the outliers and extreme values deviating significantly from the mean by over three times the standard deviation for each parameter. Subsequently, SPSS 20.0 was employed to analyze the behavioral and ERP data. For the behavior data, a 3 (session: control vs. low-intensity exercise vs. moderate-intensity exercise) × 3 (picture type: high-calorie food picture vs. low-calorie food picture vs. neutral picture) repeated measures ANOVA was conducted. Furthermore, 3 (session: control vs. low-intensity exercise vs. moderate-intensity exercise) × 2 (task condition: Go vs. No-go) × 3 (picture type: high-calorie food picture vs. low-calorie food picture vs. neutral picture) repeated measures ANOVA analyses were performed for the N2 and P3 amplitudes, respectively. *Greenhouse–Geisser* corrections were applied when sphericity was violated due to three or more within-subject levels. Follow-up analyses utilized Bonferroni adjustments, and results included partial eta-squared (*η*^2^*_p_*), with statistical significance defined as *p* < 0.05.

## 3. Results

### 3.1. Behavioral Data

Contrary to the hypothesis, the exercise intervention did not produce a significant main effect on behavioral outcomes.

For the Go RT, the 3 × 3 repeated measures ANOVA showed no significant main effect for session [F _(2,34)_ = 0.20, *p* = 0.82, *η*^2^*_p_* = 0.01] or picture type [F _(2,34)_ = 0.61, *p* = 0.55, *η*^2^*_p_* = 0.04] or interactions between session and picture type [F _(4,68)_ = 0.50, *p* = 0.74, *η*^2^*_p_* = 0.03].

Regarding the Go accuracy, the 3 × 3 repeated measures ANOVA also found no significant main effect for session [F _(2,34)_ = 2.15, *p* = 0.16, *η*^2^*_p_* = 0.11] or picture type [F _(2,34)_ = 0.78, *p* = 0.41, *η*^2^*_p_* = 0.04] or interactions between session and picture type [F _(4,68)_ = 0.15, *p* = 0.80, *η*^2^*_p_* = 0.01].

For the No-go accuracy, the 3 × 3 repeated measures ANOVA revealed a significant main effect for picture type [F _(2,34)_ = 15.74, *p* < 0.001, *η*^2^*_p_* = 0.48], with higher accuracy in the neutral picture type relative to the high-calorie (92.30 ± 1.20% vs. 87.60 ± 1.70%, *p* = 0.001) and the low-calorie food picture type (92.30 ± 1.20% vs. 85.20 ± 2.30%, *p* = 0.001), respectively; no significant difference between the high-calorie and low-calorie food picture types was found (*p* = 0.10). Furthermore, no significant main effect for session [F _(2,34)_ = 0.53, *p* = 0.59, *η*^2^*_p_* = 0.03] or interactions between session and picture type [F _(2,34)_ = 0.12, *p* = 0.91, *η*^2^*_p_* = 0.01] were observed. Means and standard deviations for reaction time and accuracy by session, task condition, and picture type are reported in Table 3.

### 3.2. EEG Data

#### 3.2.1. N2 Component

**Figure 2:**
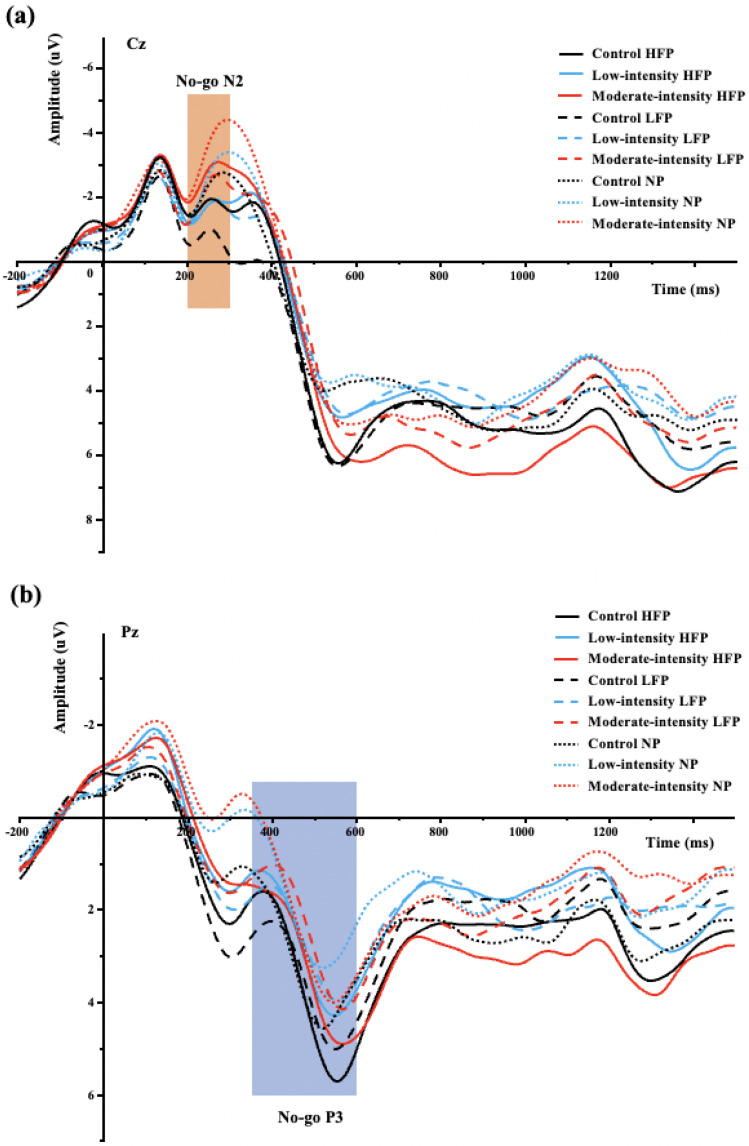
(**a**) No−go N2 average amplitudes at Cz site and (**b**) No−go P3 average amplitudes at Pz site across task conditions and picture types for the food−related Go/No−go task following moderate−intensity, low−intensity exercise, and control session. HFP = high−calorie food picture; LFP = low−calorie food picture; NP = neutral picture.

In line with the hypothesis, a significant effect of the exercise intervention on N2 outcomes was observed, with a single session of aerobic exercise resulting in alterations to N2. The 3 × 2 × 3 repeated measures ANOVA revealed a significant main effect for session [F _(2,34)_ = 4.68, *p* = 0.02, *η*^2^*_p_* = 0.22], with larger N2 amplitude following the moderate-intensity exercise (MIE) than the control session (CON) (−2.58 ± 0.86 μV vs. −1.47 ± 0.77 μV, *p* = 0.02), but with no significant difference between the low-intensity exercise (LIE) and the CON (*p* = 1.00) or the MIE and LIE (*p* = 0.16), respectively. A significant main effect for the task condition was also observed [F _(1,17)_ = 6.64, *p* = 0.02 *η*^2^*_p_* = 0.28], with the No-go condition eliciting greater N2 amplitude than the Go condition (−2.16 ± 0.81 μV vs. −1.70 ± 0.76 μV, *p* = 0.02). Additionally, there was also a significant main effect for picture type [F _(2,34)_ = 11.11, *p* < 0.001, *η*^2^*_p_* = 0.40], with a larger N2 amplitude in the neutral picture type compared with the high-calorie (−2.56 ± 0.73 μV vs. −1.73 ± 0.82 μV, *p* = 0.01) and low-calorie picture type (−2.56 ± 0.73 μV vs. −1.50 ± 0.83 μV, *p* < 0.01), respectively, but with no significant difference between the high-calorie and the low-calorie picture type (*p* = 0.80). No significant interactions between session and task condition [F _(2,34)_ = 0.27, *p* = 0.77, *η*^2^*_p_* = 0.02] or session and picture type [F _(4,68)_ = 0.70, *p* = 0.59, *η^2^_p_* = 0.04] or task condition and picture type [F _(2,34)_ = 0.50, *p* = 0.61, *η*^2^*_p_* = 0.03] or the interaction among the three factors [F _(4,68)_ = 1.05, *p* = 0.37, *η*^2^*_p_* = 0.06] were found for N2 amplitude, respectively. Table 4 displays the N2 and P3 data, while Figure 2 shows the waveforms.

#### 3.2.2. P3 Component

In contrast to the hypothesis, no main effect of the exercise intervention on P3 amplitudes was found, as a single session of aerobic exercise did not lead to any significant changes in P3 amplitudes. Regarding the P3 amplitude, the 3 × 2 × 3 repeated measures ANOVA showed no significant main effect for the session [F _(2,34)_ = 1.05, *p* = 3.62, *η*^2^*_p_* = 0.06] or task condition [F _(1,17)_ = 1.45, *p* = 0.25, *η*^2^*_p_* = 0.08] or picture type [F _(2,34)_ = 2.34, *p* = 0.11, *η^2^_p_* = 0.12]. In addition, there were no significant interactions between session and task condition [F _(2,34)_ = 0.67, *p* = 0.08, *η*^2^*_p_* = 0.14] or session and picture type [F _(4,68)_ = 0.02, *p* = 0.99, *η*^2^*_p_* = 0.00] or task condition and picture type [F _(2,34)_ = 0.18, *p* = 0.84, *η*^2^*_p_* = 0.01] or the interactions among the three factors [F _(4,68)_ = 0.60, *p* = 0.66, *η*^2^*_p_* = 0.03] for P3 amplitude, respectively.

## 4. Discussion

This study explored how acute aerobic exercise at varying intensities with a 30 min session affects both general and food-related inhibitory function on behavior and neuroelectric levels among individuals with obesity. To the best of the authors’ knowledge, this is the initial investigation into the effects of acute aerobic exercise of different intensities on general and food-related inhibitory function, as well as N2 and P3 components elicited by a food-specific Go/No-go task among young male adults with obesity. The current study found that the moderate-intensity exercise elicited larger N2 amplitude but not P3 amplitude than the control session, whereas no main effect of the session was found on behavior indicators (Go RT, Go accuracy, No-go accuracy). Additionally, food stimuli (high-calorie and low-calorie food) induced lower No-go accuracy and smaller N2 amplitude than the neutral stimuli. These findings implied that a bout of moderate-intensity exercise might influence general and food-related inhibitory function in obese individuals at the neuroelectric stage, potentially by enhancing attentional resources for managing cognitive control and conflict detection. Additionally, the reduced N2 amplitudes and No-go accuracy elicited by foods than non-foods suggested decreased ability or efficacy in allocating attentional and neural resources to manage food-related conflicts.

Regarding the behavioral data, in contrast to the hypothesis, there was no significant main effect of the session on go reaction time, go accuracy, and no-go accuracy, suggesting that acute moderate or low-intensity aerobic exercise may not enhance inhibitory control in behavior levels among individuals with obesity regarding food and non-food cues. In accordance with these results, several previous studies found no enhancement in general or food-related inhibitory function following a session of moderate-intensity aerobic exercise among healthy-weight and obese individuals [11,23,24]. For example, Vincent [23] reported that after a bout of moderate-intensity aerobic exercise, there was no improvement in the behavioral outcome on the Go/No-Go and Stroop tasks among overweight and obese adults with type 2 diabetes. Another study by Wen and Tsai [24] demonstrated that incorporating moderate-intensity aerobic exercise with resistance training did not alter Stroop task behavioral outcomes in obese individuals, but significantly influenced ERP metrics. Moreover, Bailey et al. [11] reported no significant behavior change in a food-related go/no-go task after a bout of moderate-intensity exercise (35% VO_2max_) in healthy-weight adults, but with improvement in behavior and ERP metrics after high-intensity (70% VO_2max_) exercise. While certain investigations have demonstrated the positive impacts of acute aerobic exercise on inhibitory function [17,20,27], the above studies have not confirmed this connection [11,23,24]. These findings suggest that acute moderate-intensity exercise may not have a meaningful impact on behavioral performance among obese individuals, and the observed differences could arise from variations in exercise settings, such as exercise intensities, modalities, or durations. For example, Bailey et al. [11] observed that acute high-intensity but not moderate-intensity exercise could facilitate behavior and ERP measures in food-specific inhibitory function; therefore, it was speculated that higher-intensity exercise might be more effective in modulating inhibitory and cognitive control. Previous studies have provided partial evidence supporting this claim, such as the observation that a single session of high-intensity interval exercise could improve inhibitory control over general [37] and food-related stimuli [36] in obese male adults. This improvement was reflected in faster reaction times and enhanced P3 and LPP amplitudes, indicating increased cognitive resource allocation for general and food-related inhibitory control processing [36]. Additionally, it was found that both high-intensity interval exercise and high-intensity continuous exercise could effectively promote general inhibitory control in overweight and obese children [26]. Moreover, Tsukamoto [61] reported that high-intensity interval exercise leads to greater arousal levels compared to moderate-intensity continuous exercise. According to Kahneman [62], increased arousal from exercise may boost resource allocation for effective inhibitory control. Considering the inhibitory deficits and difficulties in cognitive resource recruitment observed in obese individuals [3,14,15,16,63], it was speculated that higher-intensity exercise may elevate arousal levels, thereby enhancing resource recruitment to improve inhibitory control at both the behavioral and neuroelectric levels in individuals with obesity. Further research is needed to explore the effects and underlying mechanisms of exercise on food-related cognition in obesity.

The most important result was that in line with the hypothesis: increased N2 amplitudes were found following moderate-intensity exercise than the control session across the picture types or the task conditions, with no significant difference between the low- and moderate-intensity exercise or the low-intensity exercise and the control session. These results were in accordance with previous studies that found that acute moderate-intensity exercise could elicit larger N2 amplitudes [35,39,40,64]. These studies employed similar exercise interventions, encompassing similar intensity and duration. Noteworthy, some of these studies focused on participants exhibiting inhibitory deficits. For instance, Wang [39,40] explored methamphetamine-dependent patients, while Yu et al. [64] investigated individuals with ADHD. Previous research has identified that obese individuals share similarities with substance abuse patients and those with ADHD, as inhibitory control deficits and reduced N2 amplitudes are manifested [3,14,15,16,40,63,65,66]. The increased N2 amplitude observed in our study may suggest that a single session of moderate-intensity aerobic exercise could enhance attentional resource recruitment to support inhibitory control and conflict monitoring on food and non-food in obese adults [32,34,67]. Additionally, the study found that food items (high- and low-calorie) elicited smaller N2 amplitudes than non-food items across different sessions when accompanied by a lower No-go accuracy in food images than neutral images regardless of different sessions. N2 waveforms signify sensory processing, response inhibition, and conflict monitoring [32,34]. Previous studies demonstrated that individuals with obesity and overweight tendencies displayed diminished N2 amplitudes in food-related inhibitory tasks than counterparts with healthy weight, which indicated deficits in mobilizing accessible neural resources for effective cognitive control [14,15,16,68]. Therefore, the reduced N2 amplitudes elicited by foods suggest decreased ability or efficacy in allocating attentional and neural resources to manage food-related conflicts [14,68], which is also supported by the decreased No-go accuracy toward food than neutral items.

In terms of P3 amplitudes, in contrast to the hypothesis, no significant main effects or interactions for sessions were found, with no significant P3 change after the exercise session than in the control session. Previous investigations into the P3 amplitude after acute aerobic exercise have produced mixed findings. Some studies indicated that acute aerobic exercise could enhance the amplitude of the P3 components, which have predominantly focused on healthy-weight individuals engaged in tasks involving general inhibition [19,27,69,70]. Conversely, others have reported that acute exercise fails to augment P3 amplitude. For instance, investigations into groups such as obese individuals or those with inhibition deficits have shown no significant changes in general inhibition tasks following acute aerobic exercise [37,39,64]. Further exploration into obese individuals has revealed that following a moderate-intensity aerobic exercise session, there was no significant change in P3 amplitude during food-related inhibitory tasks, while after engaging in high-intensity exercise, P3 amplitude increased, suggesting that higher-intensity exercise may be essential for effectively modulating cognitive control and inhibition in this population [11,36]. Additionally, studies employing food-related attentional tasks (e.g., food oddball tasks and passive food viewing tasks) have not revealed a significant exercise-induced main effect on P3 amplitude but observed a significant P3 difference between food stimuli versus non-food stimuli following exercise among obese individuals other than lean counterparts, which may suggest a potential role of acute exercise in attenuating neural responses associated with food motivation [29,30]. Potential factors contributing to these discrepancies include variations in the dose–response of exercise including exercise intensity, differences in task designs, as well as participant characteristics such as obesity status and age [28,33,70], highlighting the complex relationship between acute exercise, cognitive function, and neural responses to food stimuli. Subsequent research needs rigorous control for these variables to accurately delineate how acute exercise impacts food cognition in individuals with obesity and its underlying mechanisms.

## 5. Limitations of the Study and Recommendations for Future Research

The strength of the study lies in its novel investigation into how acute aerobic exercise at varying intensities affects both general and food-related cognition, as well as the associated cognitive neural mechanisms in obese adults. The study also has some limitations. First, the sample size in the study was relatively small and only included male subjects to reduce possible gender-related effects, as previous research has shown variations in the cognitive and neural response to food-related stimuli between genders [41,42]. For example, females with food addiction exhibit a greater reliance on emotional overeating and show reduced cognitive control and homeostatic processing compared to males [42]. This behavioral pattern aligns with evidence of heightened neural activation in brain regions related to craving and taste processing, such as the anterior insula, observed in females when exposed to food-related stimuli [71]. Additionally, the N2 component is influenced by emotional valence, with women exhibiting heightened sensitivity to this effect [72,73]. Previous studies investigating N2 amplitude variations during food-related inhibition tasks [14,16] may reflect underlying gender-related differences in emotional and cognitive processing, underscoring the need for further research to disentangle these mechanisms. Upcoming studies need to include both genders, employ more extensive sample sizes, and perform comparative analyses to enhance the understanding of cognitive function and neural mechanisms linked to food in obese individuals. Second, based on the preliminary trials, obese participants were unable to maintain high-intensity continuous exercise for 20 min. Consequently, our study only utilized low and moderate-intensity continuous exercise interventions. Recent research suggests that high-intensity interval exercise (HIIE) is more feasible for obese individuals and may significantly enhance inhibitory function in this population [36,37,74]. Therefore, future research is suggested to compare HIIE with the aforementioned aerobic exercise modalities and integrate these approaches to identify the most effective interventions for improving inhibitory function in obese individuals. Lastly, this study assessed inhibitory function and neural response to food after exercise but did not measure appetite and food intake. Investigating whether acute aerobic exercise can influence the neural response to food, decrease appetite, and reduce actual food intake in obese individuals is crucial for weight loss and mitigating obesity-related cognitive impairments and physiological issues. This can be achieved through interdisciplinary approaches and multimodal technologies, such as integrating various brain imaging techniques (e.g., EEG, fMRI, fNIRS) with physiological, olfactory, and taste assessments to better understand the mechanisms of exercise-induced weight loss in obesity.

## 6. Conclusions

Consistent with the hypothesis, a single session of moderate-intensity aerobic exercise led to an increase in N2 amplitude. However, contrary to expectations, no significant effects were observed on P3 amplitude or behavioral outcomes, including Go reaction time (RT), Go accuracy, and No-go accuracy. These findings suggest that acute moderate-intensity exercise may affect general and food-related inhibitory functions in obese individuals at the neuroelectric level, potentially by enhancing attentional resources for managing cognitive control and conflict detection. Moreover, reduced N2 amplitudes and No-go accuracy in response to food stimuli compared to non-food stimuli indicate a diminished ability to allocate attentional and neural resources to manage food-related conflicts. However, due to the relatively small sample size, caution is advised when generalizing these findings to the broader population. The pilot test indicated that obese participants had difficulty sustaining high-intensity exercise at 80–90% of their maximum heart rate for a continuous or 20 min period, highlighting potential challenges in exercise adherence at high intensities within this population. Future research is needed to utilize interdisciplinary approaches and multimodal technologies to clarify how exercise influences food-related cognition, appetite regulation, and brain mechanisms in obesity, aiming to better contribute to the prevention and treatment of obesity.

## Figures and Tables

**Table 1:** Demographic characteristics (N = 18).

Variables	Mean ± SD
Age (years)	24.50 ± 5.13
Height (m)	1.76 ± 0.05
Weight (kg)	107.30 ± 14.15
BMI (kg/m^2^)	34.60 ± 4.21
Education level (years)	15.44 ± 2.12
VO_2peak_ (mL/kg/min)	30.02 ± 10.78
DEBQ	
Restrained eating	2.95 ± 0.52
Emotional eating	2.32 ± 1.09
External eating	3.47 ± 0.62

Note. BMI = Body Mass Index. The typical duration of the Chinese educational system: primary and junior high school (9 years), senior high school (3 years), and undergraduate education (4 years). All participants had completed at least senior high school, with the highest level being post-graduate. VO_2peak_ represents the participants’ cardiorespiratory fitness, as measured by the YMCA submaximal ergometer exercise test [37,49]. This test is considered safe and well-tolerated by individuals with obesity, reducing the risks associated with maximal exercise testing, and has been used in previous obesity-related studies [50,51,52]. The Dutch Eating Behavior Questionnaire (DEBQ) [53] is a widely used scale for assessing eating styles and is applicable to both obese and normal-weight populations. As the present study focuses on food-related inhibitory control, participants’ eating styles were also measured to provide a reference for future study.

**Table 2:** The numbers of analyzed EEG segments for the food-related Go/No-go task by sessions, task conditions, and picture types.

Variable	Moderate-Intensity	Low-Intensity	Control
Go HFP	148.17 ± 22.74	149.28 ± 16.96	155.17 ± 7.46
Go LFP	141.44 ± 28.74	154.61 ± 6.32	152.78 ± 12.74
Go NP	149.67 ± 15.34	155.22 ± 5.09	152.72 ± 10.50
No-go HFP	65.83 ± 12.51	70.22 ± 6.97	68.33 ± 10.31
No-go LFP	67.89 ± 8.65	68.00 ±10.02	68.17 ± 7.52
No-go NP	72.61 ± 5.54	71.83 ± 8.54	74.22 ± 5.31

Note. Values are means and standard deviations. HFP = high-calorie food picture; LFP = low-calorie food picture; NP = neutral picture.

**Table 3:** Reaction time, accuracy (%) across task conditions, and picture types for the food-related Go/No-go task following moderate-intensity exercise, low-intensity exercise, and control sessions.

Variable	Moderate-Intensity	Low-Intensity	Control
RT (ms)			
Go HFP	496.73 ± 32.53	490.90 ± 46.09	503.36 ± 55.74
Go LFP	494.07 ± 45.04	492.10 ± 54.43	495.68 ± 60.86
Go NP	493.64 ± 42.34	499.16 ± 44.93	502.28 ± 59.23
Accuracy (%)			
Go HFP	92.61 ± 14.29	96.33 ± 4.26	96.89 ± 4.58
Go LFP	94.00 ± 10.92	96.61 ± 3.99	97.78 ± 2.94
Go NP	93.72 ± 9.53	97.17 ± 3.15	97.11 ± 4.58
No-go HFP	86.94 ± 10.72	87.83 ± 8.60	88.11 ± 7.45
No-go LFP	84.72 ± 11.02	85.22 ± 12.47	85.56 ± 9.52
No-go NP	90.94 ± 6.88	92.89 ± 5.06	93.17 ± 6.44

Note. Values are means and standard deviations. HFP = high-calorie food picture; LFP = low-calorie food picture; NP = neutral picture.

**Table 4:** N2 and P3 components across task conditions and picture types for the food-related Go/No-go task following moderate-intensity exercise, low-intensity exercise, and control sessions.

Variable	Moderate-Intensity	Low-Intensity	Control
N2 (μV)			
Go HFP	−2.32 ± 3.83	−1.05 ± 3.67	−0.82 ± 3.67
Go LFP	−1.61 ± 4.32	−1.12 ± 3.66	−1.19 ± 3.79
Go NP	−3.04 ± 3.33	−2.24 ± 2.94	−1.93 ± 3.10
No-go HFP	−2.70 ± 4.12	−1.68 ± 3.47	−1.81 ± 3.63
No-go LFP	−2.37 ± 3.90	−1.87 ± 4.20	−0.86 ± 3.26
No-go NP	−3.44 ± 3.98	−2.50 ± 3.94	−2.22 ± 3.01
P3 (μV)			
Go HFP	2.92 ± 2.66	3.52 ± 3.21	3.78 ± 2.35
Go LFP	3.04 ± 2.89	3.15 ± 2.95	3.48 ± 2.58
Go NP	2.63 ± 2.52	3.08 ± 2.71	3.17 ± 2.37
No-go HFP	3.14 ± 2.96	2.79 ± 2.89	3.70 ± 3.39
No-go LFP	2.42 ± 3.25	2.70 ± 2.71	3.54 ± 3.06
No-go NP	2.34 ± 2.74	2.08 ± 2.23	3.20 ± 2.43

Note. Values are means and standard deviations. HFP = high-calorie food picture; LFP = low-calorie food picture; NP = neutral picture.

## Data Availability

The data are not publicly available due to the dataset contains information that requires additional review and preparation before it can be made publicly available.

## Appendix A

Image numbers used in the food-related Go/No-Go task from the food-pics database [54].

High-calorie food pictures: 1, 2, 6, 12, 22, 23, 34, 41, 42, 43, 45, 46, 53, 56, 57, 60, 69, 77, 79, 80, 86, 87, 89, 91, 103, 105, 115, 127, 136, 137, 140, 148, 151, 159, 167, 169, 171, 176, 185, 287, 295, 296, 297, 298, 309, 310, 313, 318, 319, 363, 387, 469, 485, 489, 507, 517, 533, 538, 547, 555.

Low-calorie food pictures: 195, 196, 197, 201, 215, 219, 228, 229, 232, 239, 242, 246, 249, 250, 252, 253, 258, 259, 262, 264, 265, 266, 267, 272, 274, 275, 277, 288, 299, 303, 305, 332, 333, 334, 335, 359, 361, 362, 368, 370, 401, 405, 411, 414, 417, 418, 424, 430, 432, 442, 448, 449, 461, 482, 490, 502, 520, 522, 529, 558.

Neutral pictures: 1004, 1005, 1007, 1008, 1009, 1010, 1013, 1014, 1028, 1030, 1031, 1032, 1033, 1035, 1038, 1056, 1060, 1134, 1140, 1141, 1142, 1143, 1146, 1149, 1151, 1153, 1156, 1208, 1210, 1212, 1213, 1214, 1218, 1219, 1223, 1224, 1225, 1227, 1231, 1234, 1235, 1239, 1240, 1242, 1243, 1244, 1249, 1250, 1252, 1256, 1258, 1261, 1265, 1267, 1268, 1272, 1274, 1276, 1277, 1279.

## References

[1] LeBlanc E.S., Patnode C.D., Webber E.M., Redmond N., Rushkin M., O’Connor E.A. (2018). Behavioral and pharmacotherapy weight loss interventions to prevent obesity-related morbidity and mortality in adults: Updated evidence report and systematic review for the us preventive services task force. JAMA.

[2] Wang Y., Zhao L., Gao L., Pan A., Xue H. (2021). Health policy and public health implications of obesity in China. Lancet Diabetes Endocrinol..

[3] Yang Y., Shields G.S., Guo C., Liu Y. (2018). Executive function performance in obesity and overweight individuals: A meta-analysis and review. Neurosci. Biobehav. Rev..

[4] Kamijo K., Pontifex M.B., Khan N.A., Raine L.B., Scudder M.R., Drollette E.S., Evans E.M., Castelli D.M., Hillman C.H. (2014). The negative association of childhood obesity to cognitive control of action monitoring. Cereb. Cortex.

[5] Prickett C., Brennan L., Stolwyk R. (2015). Examining the relationship between obesity and cognitive function: A systematic literature review. Obes. Res. Clin. Pract..

[6] Smith E., Hay P., Campbell L., Trollor J.N. (2011). A review of the association between obesity and cognitive function across the lifespan: Implications for novel approaches to prevention and treatment. Obes. Rev..

[7] García-García I., Morys F., Michaud A., Dagher A. (2020). Food addiction, skating on thin ice: A critical overview of neuroimaging findings. Curr. Addict. Rep..

[8] Lavagnino L., Arnone D., Cao B., Soares J.C., Selvaraj S. (2016). Inhibitory control in obesity and binge eating disorder: A systematic review and meta-analysis of neurocognitive and neuroimaging studies. Neurosci. Biobehav. Rev..

[9] Li G., Hu Y., Zhang W., Wang J., Ji W., Manza P., Volkow N.D., Zhang Y., Wang G.J. (2023). Brain functional and structural magnetic resonance imaging of obesity and weight loss interventions. Mol. Psychiatry.

[10] García-García I., Narberhaus A., Marqués-Iturria I., Garolera M., Rădoi A., Segura B., Pueyo R., Ariza M., Jurado M.A. (2013). Neural responses to visual food cues: Insights from functional magnetic resonance imaging. Eur. Eat. Disord. Rev..

[11] Bailey B.W., Muir A.M., Bartholomew C.L., Christensen W.F., Carbine K.A., Marsh H., LaCouture H., McCutcheon C., Larson M.J. (2021). The impact of exercise intensity on neurophysiological indices of food-related inhibitory control and cognitive control: A randomized crossover event-related potential (ERP) study. Neuroimage.

[12] Balodis I.M., Molina N.D., Kober H., Worhunsky P.D., White M.A., Sinha R., Grilo C.M., Potenza M.N. (2013). Divergent neural substrates of inhibitory control in binge eating disorder relative to other manifestations of obesity. Obesity.

[13] Hege M.A., Stingl K.T., Kullmann S., Schag K., Giel K.E., Zipfel S., Preissl H. (2015). Attentional impulsivity in binge eating disorder modulates response inhibition performance and frontal brain networks. Int. J. Obes..

[14] Liu Y., Gao X., Zhao J., Zhang L., Chen H. (2020). Neurocognitive correlates of food-related response inhibition in overweight/obese adults. Brain Topogr..

[15] Wang J., Wang H., Yu H., Wang J., Guo X., Tong S., Bao Y., Hong X. (2022). Neural mechanisms of inhibitory control deficits in obesity revealed by P3 but not N2 event-related potential component. Appetite.

[16] Wang K., Xu L., Huang T., Meng F., Yang Q., Deng Z., Chen Y., Chen G., Wang P., Qian J. (2024). Food-related inhibitory control deficits in young male adults with obesity: Behavioral and ERP evidence from a food-related go/no-go task. Physiol. Behav..

[17] Chu C.-H., Alderman B.L., Wei G.X., Chang Y.-K. (2015). Effects of acute aerobic exercise on motor response inhibition: An ERP study using the stop-signal task. J. Sport Health Sci..

[18] Chang Y.-K., Labban J.D., Gapin J.I., Etnier J.L. (2012). The effects of acute exercise on cognitive performance: A meta-analysis. Brain Res..

[19] Drollette E.S., Scudder M.R., Raine L.B., Moore R.D., Saliba B.J., Pontifex M.B., Hillman C.H. (2014). Acute exercise facilitates brain function and cognition in children who need it most: An ERP study of individual differences in inhibitory control capacity. Dev. Cogn. Neurosci..

[20] Hsieh S.-S., Huang C.-J., Wu C.-T., Chang Y.-K., Hung T.-M. (2018). Acute exercise facilitates the N450 inhibition marker and P3 attention marker during stroop test in young and older adults. J. Clin. Med..

[21] Etnier J.L., Salazar W., Landers D.M., Petruzzello S.J., Han M., Nowell P. (1997). The influence of physical fitness and exercise upon cognitive functioning: A meta-analysis. J. Sport Exerc. Psychol..

[22] Raine L.B., Kao S.C., Drollette E.S., Pontifex M.B., Pindus D., Hunt J., Kramer A.F., Hillman C.H. (2020). The role of BMI on cognition following acute physical activity in preadolescent children. Trends Neurosci. Educ..

[23] Vincent C., Hall P. (2017). Cognitive effects of a 30-min aerobic exercise bout on adults with overweight/obesity and type 2 diabetes. Obes. Sci. Pract..

[24] Wen H.-J., Tsai C.-L. (2020). Effects of acute aerobic exercise combined with resistance exercise on neurocognitive performance in obese women. Brain. Sci..

[25] Flack K.D., Anderson III R.E., McFee K.F., Kryscio R., Rush C.R. (2022). Exercise increases attentional bias towards food cues in individuals classified as overweight to obese. Physiol. Behav..

[26] Zhang L., Chu C.-H., Liu J.-H., Chen F.-T., Nien J.-T., Zhou C., Chang Y.-K. (2020). Acute coordinative exercise ameliorates general and food-cue related cognitive function in obese adolescents. J. Sports. Sci..

[27] Chang Y.-K., Alderman B.L., Chu C.-H., Wang C.-C., Song T.-F., Chen F.-T. (2017). Acute exercise has a general facilitative effect on cognitive function: A combined ERP temporal dynamics and BDNF study. Psychophysiology.

[28] Kao S.-C., Chen F.-T., Moreau D., Drollette E.S., Amireault S., Chu C.-H., Chang Y.-K. (2022). Acute effects of exercise engagement on neurocognitive function: A systematic review and meta-analysis on P3 amplitude and latency. J. Sport Exerc. Psychol..

[29] Fearnbach S.N., Silvert L., Keller K.L., Genin P.M., Morio B., Pereira B., Duclos M., Boirie Y., Thivel D. (2016). Reduced neural response to food cues following exercise is accompanied by decreased energy intake in obese adolescents. Int. J. Obes..

[30] Fearnbach S.N., Silvert L., Pereira B., Boirie Y., Duclos M., Keller K.L., Thivel D. (2017). Reduced neural responses to food cues might contribute to the anorexigenic effect of acute exercise observed in obese but not lean adolescents. Nutr. Res..

[31] Hanlon B., Larson M.J., Bailey B.W., Lecheminant J.D. (2012). Neural response to pictures of food after exercise in normal-weight and obese women. Med. Sci. Sports Exerc..

[32] Carbine K.A., Rodeback R., Modersitzki E., Miner M., Lecheminant J.D., Larson M.J. (2018). The utility of event-related potentials (ERPs) in understanding food-related cognition: A systematic review and recommendations. Appetite.

[33] Gusatovic J., Gramkow M.H., Hasselbalch S.G., Frederiksen K.S. (2022). Effects of aerobic exercise on event-related potentials related to cognitive performance: A systematic review. PeerJ.

[34] Folstein J.R., Van Petten C. (2008). Influence of cognitive control and mismatch on the N2 component of the ERP: A review. Psychophysiology.

[35] Ligeza T.S., Maciejczyk M., Kałamała P., Szygula Z., Wyczesany M. (2018). Moderate-intensity exercise boosts the N2 neural inhibition marker: A randomized and counterbalanced ERP study with precisely controlled exercise intensity. Biol. Psychol..

[36] Xie C., Alderman B.L., Meng F., Chen Y.-C., Chang Y.-K., Wang K. (2024). Acute high-intensity interval exercise improves food-related cognition in young adults with obesity: An ERP study. Int. J. Clin. Health Psychol..

[37] Xie C., Alderman B.L., Meng F., Ai J., Chang Y.-K., Li A. (2020). Acute high-intensity interval exercise improves inhibitory control among young adult males with obesity. Front. Psychol..

[38] Sardjoe M., Aldred S., Adam T., Plasqui G., Brunstrom J.M., Dourish C.T., Higgs S. (2024). Inhibitory control mediates the effect of high intensity interval exercise on food choice. Appetite.

[39] Wang D., Zhou C., Chang Y.-K. (2015). Acute exercise ameliorates craving and inhibitory deficits in methamphetamine: An ERP study. Physiol. Behav..

[40] Wang D., Zhou C., Zhao M., Wu X., Chang Y.-K. (2016). Dose–response relationships between exercise intensity, cravings, and inhibitory control in methamphetamine dependence: An ERPs study. Drug Alcohol Depend..

[41] Hallam J., Boswell R.G., DeVito E.E., Kober H. (2016). Gender-related differences in food craving and obesity. Yale J. Biol. Med..

[42] Ravichandran S., Bhatt R.R., Pandit B., Osadchiy V., Alaverdyan A., Vora P., Stains J., Naliboff B., Mayer E.A., Gupta A. (2021). Alterations in reward network functional connectivity are associated with increased food addiction in obese individuals. Sci. Rep..

[43] Zsoldos I., Sinding C., Chambaron S. (2022). Using event-related potentials to study food-related cognition: An overview of methods and perspectives for future research. Brain Cogn..

[44] Faul F., Erdfelder E., Lang A.G., Buchner A. (2007). G* Power 3: A flexible statistical power analysis program for the social, behavioral, and biomedical sciences. Behav. Res. Methods.

[45] He W., Li Q., Yang M., Jiao J., Ma X., Zhou Y., Song A., Heymsfield S.B., Zhang S., Zhu S. (2015). Lower BMI cutoffs to define overweight and obesity in China. Obesity.

[46] Xu T., Zhu G., Han S. (2015). Trend of body compositions with aging among Chinese adolescents, adults and elders. J. Nutr. Health Aging.

[47] Zhou B.F. (2002). Predictive values of body mass index and waist circumference for risk factors of certain related diseases in Chinese adults—Study on optimal cut-off points of body mass index and waist circumference in Chinese adults. Biomed. Environ. Sci..

[48] Thomas S., Reading J., Shephard R.J. (1993). Revision of the Physical Activity Readness Questionnaire (PAR-Q). Can. J. Sport Sci..

[49] Golding L.A., Myers C.R., Sinning W.E. (1989). Y’s Way to Physical Fitness: The Complete Guide to Fitness Testing and Instruction.

[50] Song T.-F., Chu C.-H., Nien J.-T., Li R.-H., Wang H.-Y., Chen A.-G., Chang Y.-C., Yang K.-T., Chang Y.-K. (2022). The Association of Obesity and Cardiorespiratory Fitness in Relation to Cognitive Flexibility: An Event-Related Potential Study. Front. Hum. Neurosci..

[51] Chi L., Hung C.-L., Lin C.-Y., Song T.-F., Chu C.-H., Chang Y.-K., Zhou C. (2021). The combined effects of obesity and cardiorespiratory fitness are associated with response inhibition: An ERP study. Int. J. Environ. Res. Public Health.

[52] Song T.-F., Chi L., Chu C.-H., Chen F.T., Zhou C., Chang Y.-K. (2016). Obesity, cardiovascular fitness, and inhibition function: An electrophysiological study. Front. Psychol..

[53] Van Strien T., Frijters J.E.R., Bergers G.P.A., Defares P.B. (1986). The Dutch Eating Behavior Questionnaire (DEBQ) for assessment of restrained, emotional, and external eating behavior. Int. J. Eat. Disord..

[54] Blechert J., Meule A., Busch N.A., Ohla K. (2014). Food-pics: An image database for experimental research on eating and appetite. Front. Psychol..

[55] Kim H., Luo J., Chu S., Cannard C., Hoffmann S., Miyakoshi M. (2023). ICA’s bug: How ghost ICs emerge from effective rank deficiency caused by EEG electrode interpolation and incorrect re-referencing. Front. Signal Process.

[56] Carbine K.A., Anderson J., Larson M.J., LeCheminant J.D., Bailey B.W. (2020). The relationship between exercise intensity and neurophysiological responses to food stimuli in women: A randomized crossover event-related potential (ERP) study. Int. J. Psychophysiol..

[57] Wardle J. (1987). Eating style: A validation study of the Dutch Eating Behaviour Questionnaire in normal subjects and women with eating disorders. J. Psychosom. Res..

[58] Dickter C.L., Bartholow B.D. (2010). Ingroup categorization and response conflict: Interactive effects of target race, flanker compatibility, and infrequency on N2 amplitude. Psychophysiology.

[59] Tilinca M., Pop T.S., Bățagă T., Zazgyva A.a., Niculescu M. (2016). Obesity and Knee Arthroscopy-a Review. J. Interdiscip. Med..

[60] Norton K., Norton L., Sadgrove D. (2010). Position statement on physical activity and exercise intensity terminology. J. Sci. Med. Sport..

[61] Tsukamoto H., Suga T., Takenaka S., Tanaka D., Takeuchi T., Hamaoka T., Isaka T., Hashimoto T. (2016). Greater impact of acute high-intensity interval exercise on post-exercise executive function compared to moderate-intensity continuous exercise. Physiol. Behav..

[62] Kahneman D. (1973). Attention and Effort.

[63] Kamijo K., Pontifex M.B., Khan N.A., Raine L.B., Scudder M.R., Drollette E.S., Evans E.M., Castelli D.M., Hillman C.H. (2012). The association of childhood obesity to neuroelectric indices of inhibition. Psychophysiology.

[64] Yu C.-L., Hsieh S.-S., Chueh T.-Y., Huang C.-J., Hillman C.H., Hung T.-M. (2020). The effects of acute aerobic exercise on inhibitory control and resting state heart rate variability in children with ADHD. Sci. Rep..

[65] Torres A., Catena A., Megías A., Maldonado A., Cándido A., Verdejo-García A., Perales J.C. (2013). Emotional and non-emotional pathways to impulsive behavior and addiction. Front. Hum. Neurosci..

[66] Wiersema R., Van Der Meere J., Roeyers H., Van C.R., Baeyens D. (2006). Event rate and event-related potentials in ADHD. J. Child Psychol. Psychiatry.

[67] Carbine K.A., LeCheminant J.D., Kelley T.A., Kapila-Ramirez A., Hill K., Masterson T., Christensen E., Larson M.J. (2024). The impact of exercise on food-related inhibitory control—Do calories, time of day, and BMI matter? Evidence from an event-related potential (ERP) study. Appetite.

[68] Liu Y., Zhao J., Zhou Y., Yang R., Han B., Zhao Y., Pang Y., Yuan H., Chen H. (2022). High-Calorie Food-Cues Impair Conflict Control: EEG Evidence from a Food-Related Stroop Task. Nutrients.

[69] Kamijo K., Nishihira Y., Hatta A., Kaneda T., Wasaka T., Kida T., Kuroiwa K. (2004). Differential influences of exercise intensity on information processing in the central nervous system. Eur. J. Appl. Physiol..

[70] Hillman C.H., Snook E.M., Jerome G.J. (2003). Acute cardiovascular exercise and executive control function. Int. J. Psychophysiol..

[71] Uher R., Treasure J., Heining M., Brammer M.J., Campbell I.C. (2006). Cerebral processing of food-related stimuli: Effects of fasting and gender. Behav. Brain Res..

[72] Glaser E., Mendrek A., Germain M., Lakis N., Lavoie M.E. (2012). Sex differences in memory of emotional images: A behavioral and electrophysiological investigation. Int. J. Psychophysiol..

[73] Lithari C., Frantzidis C.A., Papadelis C., Vivas A.B., Klados M.A., Kourtidou-Papadeli C., Pappas C., Loannides A.A., Bamidis P.D. (2010). Are females more responsive to emotional stimuli? A neurophysiological study across arousal and valence dimensions. Brain Topogr..

[74] Zhang L., Wang D., Liu S., Ren F.-F., Chi L., Xie C. (2022). Effects of acute high-intensity interval exercise and high-intensity continuous exercise on inhibitory function of overweight and obese children. Int. J. Environ. Res. Public Health.

